# A conserved role for the ARC1 E3 ligase in Brassicaceae self-incompatibility

**DOI:** 10.3389/fpls.2014.00181

**Published:** 2014-05-05

**Authors:** Emily Indriolo, Daphne R. Goring

**Affiliations:** ^1^Department of Cell & Systems Biology, University of TorontoToronto, ON, Canada; ^2^Centre for the Analysis of Genome Evolution & Function, University of TorontoToronto, ON, Canada

**Keywords:** ubiquitination, cell signaling, self-incompatibility, *Arabidopsis*, Brassicaceae

## Abstract

Ubiquitination plays essential roles in the regulation of many processes in plants including pollen rejection in self-incompatible species. In the Brassicaceae (mustard family), self-incompatibility drives the rejection of self-pollen by preventing pollen hydration following pollen contact with the stigmatic surface. Self-pollen is recognized by a ligand-receptor pair: the pollen S-locus cysteine rich/S-locus protein 11 (SCR/SP11) ligand and the pistil *S* receptor kinase (SRK). Following self-pollen contact, the SCR/SP11 ligand on the pollen surface binds to SRK on the pistil surface, and the SRK-activated signaling pathway is initiated. This pathway includes the armadillo repeat containing 1 (ARC1) protein, a member of the plant U-box (PUB) family of E3 ubiquitin ligases. ARC1 is a functional E3 ligase and is required downstream of SRK for the self-incompatibility response. This mini review highlights our recent progress in establishing ARC1’s conserved role in self-pollen rejection in *Brassica* and *Arabidopsis* species and discusses future research directions in this field.

## INTRODUCTION

Plants have evolved complex signaling networks to survive their sessile existence, and protein ubiquitination underpins many of these systems. One process regulated by ubiquitination is self-incompatibility, which prevents the acceptance of self-pollen by the pistil resulting in increased genetic diversity in a population. E3 ubiquitin ligases have been implicated in two different self-incompatibility systems, the S-Ribonuclease-based self-incompatibility (Solanaceae, Rosaceae, and Plantaginaceae) and the *S* receptor kinase (SRK)-based self-incompatibility (Brassicaceae; reviewed in Hiscock and Allen, 2008; Iwano and Takayama, 2012). This mini review will focus on the role of ubiquitination in the Brassicaceae system that has been well characterized in the *Brassica* and *Arabidopsis* species. Species in the Brassicaceae have dry stigmas, and the pollen grain must receive water for hydration from the stigmatic papilla in order to germinate and grow a pollen tube (Heslop-Harrison and Shivanna, 1977). Therefore, when a pollen grain lands on the stigmatic papilla at the top of the pistil in the flower, the stigmatic papilla can determine if the pollen grain should be accepted or rejected. If a pollen grain is determined to be self-incompatible, the stigmatic papilla will reject it by blocking pollen grain hydration and pollen tube growth. Thus, pollen contact at the stigmatic surface is a major regulatory point for pollination (reviewed in Chapman and Goring, 2010).

## THE RECEPTOR-LIGAND PAIR REGULATING SELF-INCOMPATIBILITY IN THE BRASSICACEAE

Initial research in this field was conducted on *Brassica* species (*B. oleracea*, *B. rapa*, *B. napus*) with the identification of two polymorphic loci regulating self-incompatibility. The *Brassica* pollen locus encodes the Cysteine Rich/S-locus Protein 11 (SCR/SP11) protein while the *Brassica* pistil locus encodes the SRK (Schopfer et al., 1999; Cui et al., 2000; Takasaki et al., 2000; Takayama et al., 2000; Silva et al., 2001). Each specific *SCR/SP11*-*SRK* allele pair comprises a S-haplotype, whereby recognition causes the rejection of self-pollen to prevent inbreeding, and a number of different *Brassica* S-haplotypes has been identified (reviewed in Iwano and Takayama, 2012). Sequences for different S-haplotypes (*SCR/SP11* and *SRK* alleles) have subsequently been identified in other Brassicaceae species including *Arabidopsis lyrata*, *Arabidopsis halleri*, *Arabis alpina*, *Capsella grandiflora*, and a related S-locus region in *Leavenworthia alabamica* (Kusaba et al., 2001; Schierup et al., 2001; Paetsch et al., 2006; Castric et al., 2008; Boggs et al., 2009; Foxe et al., 2009; Guo et al., 2009; Tedder et al., 2011; Chantha et al., 2013). In *Brassica*, when a self-pollen grain contacts a stigmatic papilla, the SCR/SP11 ligand from the pollen coat binds to SRK, and SRK becomes autophosphorylated (Giranton et al., 2000; Kachroo et al., 2001; Takayama et al., 2001; Shimosato et al., 2007). As expected, SRK was found to bind strongest to the corresponding SCR/SP11 ligand, but could also bind weakly to other S-haplotype-encoded SCR/SP11 ligands (Kemp and Doughty, 2007; Naithani et al., 2007; Shimosato et al., 2007). There is one known negative regulator of *Brassica* SRK, Thioredoxin H-like 1 (THL1; Bower et al., 1996; Cabrillac et al., 2001; Haffani et al., 2004). THL1’s inhibition is proposed to prevent SRK from auto-activating and signaling before the recognition of SCR/SP11 at the plasma membrane (Giranton et al., 2000; Cabrillac et al., 2001; Ivanov and Gaude, 2009). After binding of SCR/SP11 to SRK, the self-incompatibility signaling cascade is initiated. This rejection is localized to the point of pollen contact, as a single papilla can simultaneously accept a compatible pollen grain and reject a self-incompatible pollen grain (Dickinson, 1995).

## REGULATORY PROTEINS ACTING DOWNSTREAM OF SRK

In addition to the role of SCR/SP11 and SRK in mediating initial self-pollen recognition, there are two other proteins that have been identified as positive regulators of the self-incompatibility response in *Brassica*: the M-locus protein kinase (MLPK; Murase et al., 2004; Kakita et al., 2007b) and the E3 ubiquitin ligase, Armadillo (ARM)-repeat containing 1 (ARC1; Gu et al., 1998; Stone et al., 1999, 2003). As well, more recent research from our group has tied the role of Exo70A1, a key component for polarized exocytosis to be negatively regulated by ARC1 in the self-incompatibility response (Samuel et al., 2009; Safavian and Goring, 2013; Indriolo et al., 2014; Safavian et al., Submitted). *B. rapa* MLPK is a Receptor-Like Cytoplasmic Kinase (RLCK) that, through alternate splicing, is localized to the plasma membrane via an N-terminal myristoylation site or an N-terminal hydrophobic region, and both forms can complement *mlpk* mutant stigmatic papillae (Murase et al., 2004; Kakita et al., 2007a). MLPK is proposed to interact with SRK at the plasma membrane, and the SRK-MLPK complex is proposed to phosphorylate downstream signaling proteins (Kakita et al., 2007a,b; Samuel et al., 2008). *A. thaliana* RLCKs that are closely related to MLPK have been identified, but a corresponding role to MLPK in *Arabidopsis* self-incompatibility has not been elucidated yet (Kakita et al., 2007a). So far, the only other known downstream component, ARC1, is a member of the Plant U-box (PUB)/ARM repeat family of E3 ligases (Mudgil et al., 2004; Samuel et al., 2006; Yee and Goring, 2009). While ARC1’s role in *B. napus* and *A. lyrata* self-incompatibility has not been disputed (Stone et al., 1999; Indriolo et al., 2012), some debate does exist as to whether ARC1 is required for reconstituting self-incompatibility in *A. thaliana* as discussed below (Indriolo et al., 2014). Part of this will likely turn out to be due to the nature of signaling systems using complex multi-branched pathways; as such, one would expect more signaling proteins to be implicated in the SRK pathway in the future.

Plant U-box-armadillo repeat E3 ligases are involved in a wide variety of plant processes including plant-microbe interactions, abiotic stress responses, hormone responses, and development (Mbengue et al., 2010; Lu et al., 2011; Salt et al., 2011; Liu et al., 2012; Seo et al., 2012; Stegmann et al., 2012; Vogelmann et al., 2012; Wang et al., 2013). Several UND-PUB-ARM E3 ligases have been found to interact with receptor kinases (Samuel et al., 2008; Mbengue et al., 2010; Lu et al., 2011). The conserved U-box domain of ~ 70 residues was originally identified in yeast UFD2 protein and interacts with the E2 conjugating enzyme (Koegl et al., 1999; Hatakeyama and Nakayama, 2003; Andersen et al., 2004; Schulman and Chen, 2005; Wiborg et al., 2008). A subset of the PUB-ARM proteins, including ARC1, contain a conserved U-box N-terminal domain (UND; Mudgil et al., 2004; Samuel et al., 2006). The UND domain is proposed to give specificity for proteins ubiquitinated by ARC1 such as the proposed target, Exo70A1, in the instance of self-incompatibility.

## ARC1 IS AN E3 LIGASE INVOLVED IN SELF-INCOMPATIBILITY SIGNALING

**FIGURE 1:**
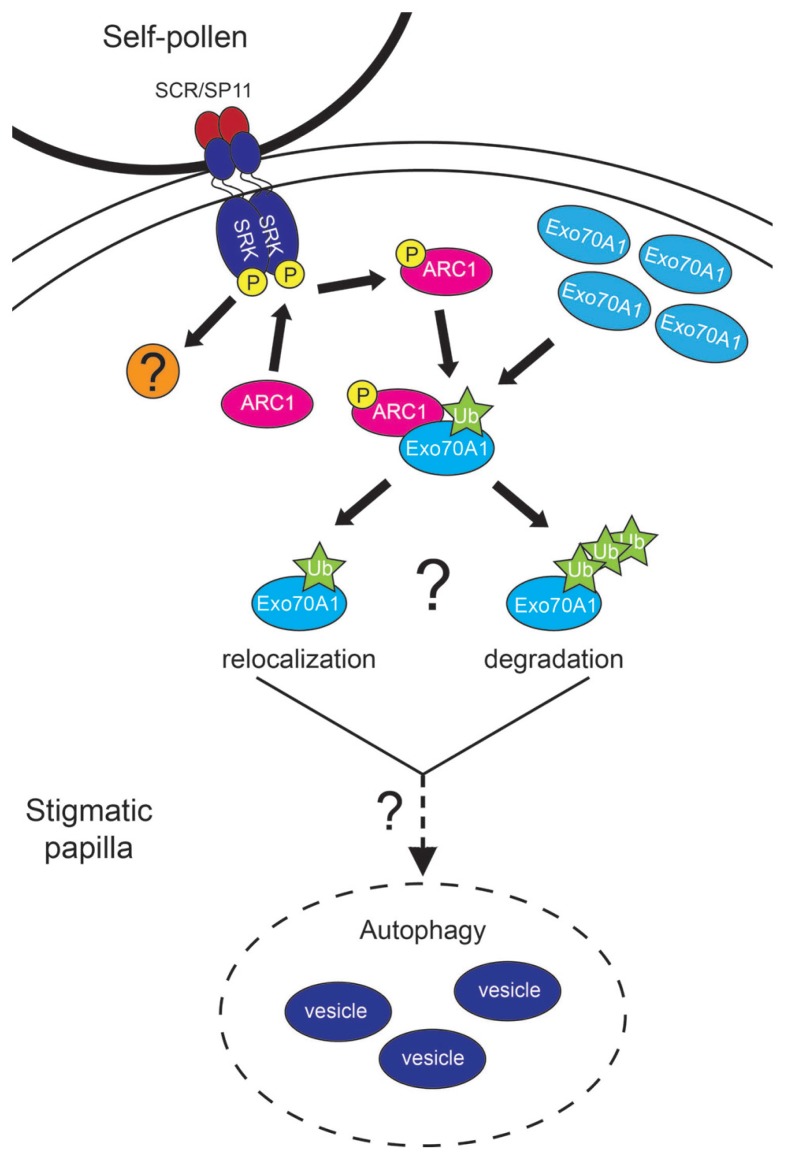
**Model for the Self-Incompatibility response in *Arabidopsis.*** Upon pollen contact with the stigmatic papilla, the papilla must determine if the pollen grain is self or non-self. For a self-pollen grain, the SCR/SP11 ligand binds to SRK, and SRK will then become phosphorylated. ARC1 interacts with the phosphorylated cytoplasmic kinase domain of SRK and become phosphorylated and activated. ARC1 then seeks its target for ubiquitination, Exo70A1, which is located at the plasma membrane in preparation for exocyst-mediated exocytosis. ARC1, with the help of an E1 activating enzyme and E2 conjugating enzyme, ubiquitinates Exo70A1. The nature of this ubiquitination is not known, as it could be either mono-ubiqitination resulting in Exo70A1 relocalization, or poly ubiquitination leading to degradation of Exo70A1 by the 26S proteosome. Either way, the inhibition of Exo70A1 appears to activate an autophagic response that leads to degradation of the secretory vesicles. There is also another (unknown) factor functioning downstream of SRK in this pathway. How ARC1, this factor, and the ubiquitination of Exo70A1 signals for autophagy is not known. Once the secretory vesicles have been removed, the self-pollen grain will not receive the resources needed for hydration and germination; as a result will be rejected.

Armadillo repeat-containing 1 was originally identified in *B. napus*, through a yeast two-hybrid screen for SRK kinase domain interactors, and ARC1 was found to bind to SRK through its ARM repeat domain (Gu et al., 1998). ARC1 can be phosphorylated by SRK, but is more strongly phosphorylated by MLPK *in vitro* (Gu et al., 1998; Samuel et al., 2008). ARC1 is composed of the three distinct protein domains described above (UND, U-box, ARM repeat domain), and has functional nuclear localization and nuclear export signals. When transiently expressed in tobacco BY2 cells, ARC1 was localized to both the cytoplasm and nucleus, shuttling back and forth between these two compartments (Stone et al., 2003). The function of ARC1’s nuclear localization is still unclear, especially as it is expected to be near the plasma membrane for its role in self-incompatibility (**Figure 1**, described in more detail below). When ARC1 was co-expressed with active SRK or MLPK kinase domains, it no longer shuttled to the nucleus suggesting that ARC1 phosphorylation alters its localization and may be important for its function in the self-incompatibility pathway (Stone et al., 2003; Samuel et al., 2008). *B. napus ARC1* displays stigma-specific expression, and the knock-down of *ARC1* expression by antisense suppression resulted in a gained self-pollen acceptance, instead of rejection, indicating a breakdown in the self-incompatible pathway (Gu et al., 1998; Stone et al., 1999). ARC1 was shown to have *in vitro* E3 ligase activity, and the importance of ubiquitination in self-incompatibility came from analyses of ubiquitinated proteins in the stigma. Wild-type *B. napus* stigmas that were pollinated with self-incompatible pollen were shown, by immunoblotting with an anti-ubiquitin antibody, to be enriched in ubiquitinated proteins (Stone et al., 2003; Samuel et al., 2011). In contrast, self-pollinated *ARC1*-antisense-suppressed stigmas had a lower level of ubiquitinated proteins. Therefore, these data suggest that the presence of ARC1 led to ubiquitinated stigma proteins following self-incompatible pollinations. Given that all this work was done in *B. napus*, an outstanding question in the field was whether ARC1’s function was conserved in other Brassicaceae species.

## ARC1 PLAYS A CONSERVED ROLE IN SELF-INCOMPATIBILITY SIGNALING ACROSS THE BRASSICACEAE

To better understand the Brassicaceae self-incompatibility pathway, one direction of research has examined this trait in *Arabidopsis* species. *A. thaliana* lost the self-incompatibility trait due to mutations in the *SCR*/*SRK* genes while another *Arabidopsis* species, *A. lyrata*, has remained naturally self-incompatible (Kusaba et al., 2001; Schierup et al., 2001). Some *A. thaliana* ecotypes, such as Wei-1 and Old-1, were actually found to carry intact *SRK* genes and exhibited self-incompatibility when pollinated with *A. halleri* pollen expressing the matching *SCR* ligand (Tsuchimatsu et al., 2010). Self-compatible *A. thaliana* has been used as an artificial system for reconstructing self-incompatibility by transforming *A. lyrata SCR* and *SRK* genes into different *A. thaliana* ecotypes (Nasrallah et al., 2004; Boggs et al., 2009; Rea et al., 2010; Tsuchimatsu et al., 2010). Interestingly, transgenic *A. thaliana* expressing *SCR* and *SRK* did not always result in the generation of self-incompatibility. Some transgenic ecotypes such as Sha, Kas-2, and C24 were reported to produce self-incompatible flowers, while other transgenic ecotypes such as Col-0, Mt-0, Nd-0, and No remained self-compatible (Nasrallah et al., 2004; Boggs et al., 2009). However, there is ambiguity in these delineations; for example, transgenic *A. thaliana* Col-0 were sometimes reported as becoming self-incompatible with *SCR* and *SRK* expression (Nasrallah et al., 2002; Kitashiba et al., 2011) and other times reported as remaining self-compatible (Nasrallah et al., 2004; Boggs et al., 2009). The ambiguity appears to stem from the types of assays used to assess self-incompatiblity, and how self-incompatibility is defined; from a very narrow temporal window (Nasrallah et al., 2002) or flower age (Tsuchimatsu et al., 2010) to perhaps a fully self-incompatible flower. The defining purpose of self-incompatibililty is to prevent inbreeding; thus, it would have been clearer if seed set was measured consistently in these studies. Nevertheless, these data suggest that there are some inherent differences between some of the *A. thaliana* ecotypes with regards to the strength of the reconstituted self-incompatibility.

*Arabidopsis* species diverged 20–40 Mya from *Brassica* species (Franzke et al., 2011), and it has been proposed that *A. thaliana* uses a different signaling pathway downstream of SRK to *Brassica* species (Rea et al., 2010; Kitashiba et al., 2011). However, to date, no candidates have been identified for this proposed pathway. Thus, we were interested in assessing the contributions of ARC1 to self-incompatibility in *Arabidopsis* species. *ARC1* was identified in the *A. lyrata* genome sequence and determined to be deleted in the *A. thaliana* Col-0 and C24 ecotypes (Kitashiba et al., 2011; Indriolo et al., 2012). *ARC1* is expressed in *A. lyrata* stigmas, but also expressed at lower levels in other tissues (Indriolo et al., 2012). Given that some ecotypes such as Wei-1 and Old-1 contained a functional *SRK* gene (Tsuchimatsu et al., 2010), we investigated whether an intact *ARC1* gene was present in any *A. thaliana* ecotypes. Despite screening 357 different *A. thaliana* ecotypes, all ecotypes carried the same deletion resulting in a non-functional *ARC1* gene. Thus, the *ARC1* deletion likely occurred before the different *SRK* and *SCR/SP11* inactivating mutations (since some ecotypes still carried functional *SCR* or *SRK* genes; Indriolo et al., 2012). Further analyses on sequenced genomes from other self-compatible Brassicaceae species revealed that in several self-compatible species, *ARC1* was non-functional due to large deletions or smaller mutations (Indriolo et al., 2012). As well, self-incompatible species carried intact copies of the *ARC1* gene. ARC1’s function was then specifically examined in self-incompatible *A. lyrata* to determine if it was necessary for the rejection of self-pollen. *ARC1* expression was knocked down by transforming an *ARC1* RNAi construct into *A. lyrata* resulting in transgenic plants that were no longer able to fully reject self-pollen when self-pollinated. In addition to the observed pollen grain adhesion and pollen tube growth, self-pollinations for transgenic *ARC1* RNAi *A. lyrata* resulted in seed set. In contrast, wild-type self-incompatible *A. lyrata* pollinations resulted in a rejection of all self-pollen and a complete absence of seeds. These results conclusively showed that *ARC1* is required for the complete rejection of self-incompatible pollen in *A. lyrata*, and supported a conserved role for ARC1 downstream of the SRK in both *Brassica* and *Arabidopsis* species (Indriolo et al., 2012). It is important to note though that these results do not preclude the presence of other downstream signaling proteins as only a partial breakdown of self-incompatibility was achieved in both studies (Stone et al., 1999; Indriolo et al., 2012).

Most recently, we have completed the reciprocal part of this research and tested ARC1’s role in *A. thaliana* by transforming *A. lyrata ARC1* and *B. napus ARC1* along with the *A. lyrata SCRb* and *SRKb* transgenes (Indriolo et al., 2014). Two ecotypes reported to have differing results when transformed with *A. lyrata SCRb* and *SRKb* were selected: Col-0, which remained self-compatible, and Sha, which became self-incompatible (Nasrallah et al., 2004; Boggs et al., 2009). The main question examined was whether transforming *ARC1*, along with *SCRb-SRKb*, into these two ecotypes resulted in a stronger self-incompatibility trait. We observed that transgenic *SCRb-SRKb* Col-0 remained self-compatible as previously reported, but the addition of *ARC1* with *SCRb-SRKb* in Col-0 resulted in clear self-incompatibility. The Sha ecotype was previously reported to display a self-incompatible phenotype with the expression of *SCRb-SRKb* alone, and we did identify some lines that displayed a moderate self-incompatibility trait. However, we found that the addition of *ARC1* resulted in a more robust and stable self-incompatible phenotype with the near complete rejection of self-pollen and little or no seed set (Indriolo et al., 2014). The expression of *ARC1* did not affect the levels of *SCRb* and *SRKb* transcripts, demonstrating that ARC1 functioned at the protein level as a direct component of the self-incompatibility signaling pathway (Indriolo et al., 2014). Interestingly, we observed an approach herkogamy trait in the transgenic *A. thaliana SCRb-SRKb-ARC1* plants, where the stigma was positioned above the anthers to avoid self-pollination. Because of this, manual pollinations were conducted for all the analyses to bypass this additional pollen avoidance trait. Overall, the results of these transgenic experiments clearly showed that the addition of either *A. lyrata ARC1* or *B. napus ARC1* with *SCRb-SRKb* led to stronger self-incompatibility phenotype in both the Col-0 and Sha ecotypes (Indriolo et al., 2014). Furthermore, both *A. lyrata* ARC1 and *B. napus* ARC1 exhibited a matching phenotype; thus despite the 20–40 Mya of divergence between *A. lyrata* and *B. napus*, the ARC1 protein retained a conserved function in self-incompatibility.

## EXO70A1, THE TARGET OF ARC1 IN THE SELF-INCOMPATIBILITY PATHWAY

To determine how ARC1 may be driving the rejection of self-pollen, a yeast two-hybrid screen was performed to search for potential targets of ubiquitination by ARC1, and Exo70A1 was identified (Samuel et al., 2009). Further experiments showed that Exo70A1, not only interacted with ARC1, but that it could be ubiquitinated by ARC1 in an *in vitro* assay (Samuel et al., 2009). So how does the ubiquitination of Exo70A1 result in the rejection of self-pollen? Before answering this question, Exo70A1’s function in the stigma for compatible pollen responses requires explanation. Exo70A1 has previously been shown to act as a marker for secretory vesicles that are delivered to a target membrane via the exocyst complex (reviewed in He and Guo, 2009; Heider and Munson, 2012; Zárský et al., 2013). In response to compatible pollen, the exocyst complex is proposed to mediate polarized exocytosis by docking secretory vesicles at the stigmatic papillar plasma membrane under the pollen contact point. Observations of RFP:Exo70A1 localized to the plasma membrane in stigmatic papillae of mature flowers supported this hypothesis; that Exo70A1 was localized where it was needed to direct exocyst assembly and delivery of secretory vesicles to facilitate hydration of the compatible pollen grain (Samuel et al., 2009). Both *A. thaliana exo70a1-1* mutant stigmas and transgenic *B. napus* plants with a stigma-expressed *Exo70A1* RNAi knockdown construct were impaired in accepting wild-type compatible pollen validating this prediction. As well, the stigma-specific expression of *RFP:Exo70A1* rescued the stigmatic defect in the *exo70a1-1* mutant (Samuel et al., 2009). Recently, Li et al. (2013) proposed that Exo70A1 has a tissue-specific expression pattern and functions in developing tracheary elements; however, these observations are not consistent with other research published on *A. thaliana* Exo70A1 (reviewed in Zárský et al., 2013). Li et al. (2013) also published that *A. thaliana exo70a1-1* mutant stigmas did not have a defect in accepting compatible pollen. With *A. thaliana* having dry stigmas and previous studies showing that pollen hydration defects can be rescued by high humidity (Preuss et al., 1993; Hülskamp et al., 1995), we tested pollinations under low and high relative humidity and found that this factor may help to explain the discrepancies between these two studies (Safavian et al., Submitted). At low relative humidity, no pollen grain acceptance or seed set was observed on the *exo70a1-1* mutant stigma as we had previously published, but high relative humidity conditions did partially rescue the *exo70a1-1* mutant stigma resulting in some compatible pollen acceptance and seed set (Safavian et al., Submitted). *Exo70A1*’s expression in the stigma was also verified by RT-PCR (Safavian et al., Submitted). The requirement of Exo70A1 in the stigmatic papillae for compatible pollen acceptance makes it an excellent target for ARC1 in the self-incompatibility pathway. If Exo70A1’s activity is inhibited through ubiquitination by ARC1, then the required secretory activity would be blocked in the stigmatic papilla at the pollen contact site causing pollen grain rejection (**Figure 1**).

To delve into the idea of secretion being required for compatible pollen acceptance and inhibited for self-incompatible pollen rejection, detailed ultrastructural TEM studies were conducted at different time points following pollinations (Safavian and Goring, 2013; Indriolo et al., 2014). In wild-type compatible pollinations at 10 minutes post-pollination, what appeared to be secretory vesicles were visible at the stigmatic papillar plasma membrane at the point of pollen contact in *A. thaliana* and *A. lyrata* (Safavian and Goring, 2013). Intriguingly, *B. napus* stigmatic papillae appeared to use multivesicular bodies (MVBs) instead for secretion at the point of pollen contact. This switch to MVBs may be connected to the presence of a thicker cell wall and perhaps a need for increased secretion (Safavian and Goring, 2013). This observed secretory activity was disrupted in the *A. thaliana exo70a1-1* mutant stigmas and the transgenic *B. napus Exo70A1* RNAi knockdown stigmas following the application of wild-type compatible pollen (Safavian and Goring, 2013). Thus, these studies indicated that Exo70A1 is necessary for exocytosis in the stigmatic papillae following compatible pollinations for promoting pollen hydration and germination.

Self-incompatible pollinations in *B. napus, A. lyrata*, and transgenic *SCRb-SRKb-ARC1 A. thaliana* all displayed signs of disrupted secretory activity (Safavian and Goring, 2013; Indriolo et al., 2014). Originally, we had thought that the outcome of disrupted secretory activity would be an accumulation of secretory vesicles/MVBs in the cytoplasm, and some of this was seen in the transgenic *SCRb-SRKb-ARC1 A. thaliana*. However, in *A. thaliana* and *A. lyrata* stigmatic papillae, autophagy appeared to be induced at 10 min post-self-incompatible pollination. That is, secretory vesicles (and cytoplasm) were being engulfed by autophagosomes and sent to the vacuole for degradation. This resulted in the presence of autophagic vacuoles and autophagic bodies in the stigmatic papillar vacuoles (Safavian and Goring, 2013; Indriolo et al., 2014). Again, *B. napus* stigmatic papillae showed a different degradation response with MVBs re-directed to the vacuole (Safavian and Goring, 2013). These observations suggest that ARC1 may promote more than just the disruption of Exo70A1-guided secretory activity in self-incompatible pollinations (**Figure 1**). Interestingly in *A. thaliana*, an ecotype specific difference was observed with the addition of *ARC1* with the *SCRb-SRKb* transgenes. The autophagic response was strongest in the transgenic *SCRb-SRKb-ARC1 A. thaliana* Sha ecotype and not as clear in the transgenic *SCRb-SRKb-ARC1 A. thaliana* Col-0 ecotype or the transgenic *SCRb-SRKb A. thaliana* Sha plants lacking *ARC1* (Indriolo et al., 2014). This autophagic response in the transgenic *SCRb-SRKb-ARC1 A. thaliana* Sha ecotype was more similar to that observed in *A. lyrata* self-incompatible pollinations, and was correlated with the strong self-incompatibility response that we observed in *A. thaliana* Sha. Finally, the disruption of vesicle secretion was also observed in the transgenic *SCRb-SRKb A. thaliana* Sha plants lacking *ARC1*, supporting the previous models that there is an additional (unknown) signaling factor(s) functioning downstream of SRK in the cellular response for self-pollen rejection (Rea et al., 2010; Indriolo et al., 2014).

## FUTURE RESEARCH DIRECTIONS

The phenotypes arising from the knockdown of *ARC1* in transgenic *ARC1* RNAi *A. lyrata* (Indriolo et al., 2012) and the addition of *ARC1* with *SCRb-SRKb* in transgenic *A. thaliana* (Indriolo et al., 2014) give strong evidence that the *SRK-ARC1* self-incompatibility signaling pathway is conserved between *Arabidopsis* and *Brassica* species. Despite the progress made into elucidating the role of ARC1 in the self-incompatibility pathway, more questions remain on how it functions as an E3 ubiquitin ligase to regulate downstream targets. Future research directions should focus on identifying the *in vivo* protein modifications of ARC1 with Exo70A1 during the self-incompatibility response. If ARC1 ubiquitinates Exo70A1, what is the outcome, redirection of Exo70A1’s localization or degradation by the 26S proteasome? Secondly, how is autophagy in *Arabidopsis* or vacuolar targeting of MVBs in *B. napus* induced as part the self-incompatibility response, and how is ARC1 and/or other factors involved in this? Finally, the other unknown signaling factor(s) functioning downstream of SRK needs to be identified and how they function in the cellular response for self-pollen rejection needs to be determined.

## AUTHOR CONTRIBUTIONS

Both Emily Indriolo and Daphne R. Goring conceived, designed and wrote this review.

## Conflict of Interest Statement

Research in DRG’s laboratory was conducted in the absence of any commercial or financial relationships that could be construed as a potential conflict of interest.
